# The Informal Care Burden of Older Adults with Disability, Dementia, and Concurrent Cardiometabolic Multimorbidity: A Qualitative Study Based on Social Ecosystem Theory

**DOI:** 10.3390/healthcare14203398

**Published:** 2026-10-12

**Authors:** Yuting Yang, Reyisaimu Wumaierjiang, Lin Xia, Xiaolong Wang, Yulong Ma, Mingxia Jing

**Affiliations:** School of Public Health, Shihezi University, Bei’er Road, Shihezi City 832003, China; yangyuting@stu.shzu.edu.cn (Y.Y.); rwumaierjiang@whu.edu.cn (R.W.); xialin@stu.shzu.edu.cn (L.X.); wangxiaolong@stu.shzu.edu.cn (X.W.)

**Keywords:** disability, dementia, cardiometabolic multimorbidity, informal care burden, social–ecological systems theory, Colaizzi’s seven-step

## Abstract

**Background**: The co-occurrence of disability, dementia, and cardiometabolic multimorbidity (CMM) creates a highly complex home-care context requiring informal caregivers to simultaneously provide daily living assistance, cognitive and behavioral support, and management of multiple chronic conditions, yet the multidimensional burden these caregivers bear remains poorly understood. **Objective**: To explore the caregiving burden and associated factors among primary informal caregivers of older adults with disability, dementia, and concurrent CMM, and to interpret them from a social–ecological systems perspective. **Methods**: A descriptive qualitative study was conducted. Purposive sampling recruited 26 primary informal caregivers from urban and surrounding rural areas of Shihezi, Xinjiang, for semi-structured interviews. All care recipients had impaired functional ability, clinically confirmed dementia, and at least two cardiometabolic diseases. Data were analyzed inductively with Colaizzi’s seven-step method, followed by interpretive mapping based on social–ecological systems theory. **Results**: At the micro level, burden mainly involved accumulated complex caregiving tasks, decision-making uncertainty, and physical and psychological exhaustion. At the mezzo level, caregiving responsibility was concentrated in individual family members, and community support was inadequately tailored to these families’ needs. At the macro level, caregivers’ use of external support was shaped by institutional accessibility, geographic distribution of care resources, and sociocultural expectations. Factors across levels interacted and jointly determined overall burden. **Conclusions**: The burden of informal caregivers of older adults with disability, dementia, and concurrent CMM is multidimensional and multilevel. Nursing and long-term care support should promote family sharing of caregiving responsibility, continuous professional support, and better family–external resource coordination to enhance the sustainability of home-based care.

## 1. Introduction

The World Health Organization estimates that the global population aged 60 years and older will increase from 1 billion in 2020 to 1.4 billion by 2030 and approximately 2.1 billion by 2050 [1]. Currently, approximately 142 million older adults worldwide are unable to independently meet their basic daily living needs, and nearly two thirds of older adults may require some degree of care and support during their lifetime [2]. According to data from the National Bureau of Statistics of China, by the end of 2024, adults aged 60 years and older accounted for 22% of the total population, while the population aged 65 years and older exceeded 220 million, accounting for 15.6% of the national population [3]. As population aging continues to accelerate, the number of older adults experiencing functional impairment and cognitive decline, together with the associated demand for long-term care, continues to increase [4]. Meeting the long-term care needs of older adults with complex health conditions has therefore become an important challenge for both public health and social care systems.

Older adults with both disability and dementia represent a population with particularly high long-term care needs. Their care extends beyond assistance with activities of daily living to include cognitive, behavioral, and safety management. The presence of comorbid chronic diseases further increases the complexity of long-term care. More than one third of adults aged 65 years and older live with two or more chronic health conditions [5]. Importantly, different combinations of chronic diseases are associated with distinct disease trajectories, health outcomes, and management needs [6]. Cardiometabolic multimorbidity (CMM) refers to the co-occurrence of cardiometabolic conditions such as hypertension, diabetes, coronary heart disease, stroke, and dyslipidemia [7]. Previous studies have demonstrated that CMM is closely associated with functional dependence [8], adverse cognitive outcomes [9,10,11], poorer health-related quality of life [12], increased healthcare utilization, and elevated mortality among older adults [13]. Because multiple organ systems may be affected simultaneously, individuals with CMM often require sustained monitoring and long-term disease management, placing additional demands on both patients and their caregivers [14,15]. Accordingly, older adults who simultaneously experience disability, dementia, and CMM constitute a particularly complex care population in whom functional dependence, cognitive and behavioral problems, and chronic disease management needs coexist and interact.

For older adults with both disability and dementia, limitations in functional independence make prolonged caregiver assistance necessary. When CMM is also present, caregiving becomes considerably more complex [16,17]. In addition to helping with eating, mobility, personal hygiene, safety, and dementia-related cognitive and behavioral problems [18], caregivers may also be required to assist with prescribed medication regimens, record health indicators such as blood pressure and blood glucose, support dietary and lifestyle management, accompany care recipients to medical appointments, and coordinate healthcare across multiple chronic conditions [19]. At the same time, dementia may impair understanding, treatment adherence, cooperation, and behavioral regulation, thereby making chronic disease management and daily care more difficult to implement consistently [16]. Thus, caregiving for this population cannot be understood simply as the addition of basic personal care to chronic disease management [20]. Rather, caregivers must continuously coordinate multiple interrelated care tasks and respond to changing care situations, placing substantial demands on their disease-management knowledge, caregiving skills, and capacity for sustained care.

Informal care remains a fundamental component of long-term care across different healthcare systems. Although countries vary considerably in formal long-term care coverage, public expenditure, and support for family caregivers, family members and other members of social networks continue to undertake a substantial proportion of long-term caregiving. Informal care refers to unpaid care and daily assistance provided outside formal employment or professional care arrangements by family members, relatives, friends, or other members of an individual’s social network, usually within home or community settings. According to OECD data published in 2025, approximately 60% of older adults with long-term care needs in the 19 countries analyzed received informal care only, indicating that informal caregiving remains widespread even in countries with relatively established formal long-term care systems [21]. China similarly relies heavily on family-based long-term care. Data from the Fifth Sample Survey on the Living Conditions of Older Adults in Urban and Rural China showed that among older adults who reported a need for assistance with daily living and actually received such assistance, spouses accounted for 45.5% of primary caregivers and children or children-in-law accounted for 47.6%, together representing 93.1% [22]. In Xinjiang, a geographically remote region in western China, population aging, outmigration of younger workers, limited market-based care resources, insufficient institutional care coverage, and the ongoing development of integrated home- and community-based health and eldercare services may further reinforce reliance on informal family caregiving. Consequently, the caregiving difficulties experienced by informal caregivers in this region warrant particular attention [23,24,25].

Despite the importance of informal caregiving, the burden experienced by caregivers of older adults who simultaneously have disability, dementia, and CMM remains insufficiently understood. International research on informal caregiver burden has gradually expanded from overall burden measurement to multidimensional mechanisms and intervention strategies. A 2025 scoping review by Liu et al. [16], which included 29 studies, found that most existing studies employed quantitative cross-sectional designs and primarily focused on overall caregiver burden and psychological health outcomes [26]. Cognitive problems and chronic disease management have often been investigated separately, leaving limited understanding of how caregivers experience and manage these demands when they occur simultaneously. In China, recent studies have examined caregiving experiences among family caregivers of people with dementia, family care support for older adults with disability and dementia, and caregiving experiences among caregivers of patients with specific conditions such as stroke [27,28,29]. However, most studies have focused either on a single disease or on older adults with disability and dementia in general. Qualitative evidence specifically concerning informal caregivers of older adults with concurrent disability, dementia, and CMM remains scarce, and the ways in which contextual factors jointly shape their caregiving burden have not been adequately explained.

Caregiver burden is influenced not only by characteristics of the caregiver and care recipient but also by family relationships, community and healthcare services, broader social resources, and policy environments. A solely individual-level perspective is therefore insufficient to explain how this burden develops and is sustained. Social–ecological systems theory emphasizes dynamic interactions between individuals and their surrounding environments and conceptualizes these influences across micro-, meso-, and macro-level systems, including individual, family, community, policy, and sociocultural factors. The theory has increasingly been applied in family caregiving and home-based dementia care research to examine how individual, interpersonal, community, and structural environments jointly shape caregiving experiences. Compared with theoretical approaches that primarily emphasize stress processes, role conflict, or social support, a social ecological systems framework provides a broader structure for examining how family resources, community services, and social environments interact in the care of older adults with disability and dementia and concurrent CMM [30,31,32].

In summary, current evidence provides an incomplete understanding of the actual caregiving burden and multilevel influencing factors experienced by informal caregivers of older adults with disability and dementia and concurrent CMM, particularly within the regional context of Xinjiang. Therefore, this study adopted a descriptive qualitative design guided by social–ecological systems theory to explore the caregiving burden and associated factors experienced by primary informal caregivers of this population and to examine interactions among individual, family, community, and broader social factors. By focusing specifically on older adults who simultaneously experience disability, clinically diagnosed dementia, and CMM, this study aims to extend qualitative evidence on informal caregiving under complex health conditions, identify contextually relevant support needs, and provide empirical evidence for the development of more appropriately targeted and stratified family- and community-based care support and long-term care services.

## 2. Materials and Methods

### 2.1. Design

This study employed a descriptive qualitative design to explore the caregiving burden and associated factors experienced by primary informal caregivers of older adults with disability and dementia and concurrent cardiometabolic multimorbidity (CMM). Descriptive qualitative research aims to remain close to participants’ original accounts and to provide a direct and systematic description of their lived care experiences, making it particularly appropriate for complex caregiving issues that have not yet been adequately understood [33].

Because this study focused on caregivers’ first-hand experiences of long-term caregiving, the procedural steps of Colaizzi’s seven-step method were used to guide the analysis so that interpretations could be continuously traced back to participants’ original accounts, thereby enhancing the systematic nature and credibility of the analysis [34].

Social–ecological systems theory was subsequently used as a second-stage interpretive framework. Themes generated inductively from the interview data were mapped onto individual, family and interpersonal, community service, and broader social-environmental levels, and the reinforcing, buffering, or constraining relationships among these levels were further examined. The theoretical framework was not used as a set of predetermined coding categories; findings that were relevant to the research question but could not be readily accommodated within predefined ecological levels were retained. This approach combined inductive analysis with theory-informed interpretation, allowing the findings to remain grounded in caregivers’ actual experiences while also examining how factors operating at different ecological levels shaped caregiving burden.

### 2.2. Participants and Sampling

#### 2.2.1. Study Setting and Sampling

Participants were recruited from urban communities and surrounding rural areas of Shihezi City, Xinjiang, China. Purposive sampling was used to identify home-dwelling older adults who simultaneously had disability, clinically diagnosed dementia, and CMM, together with their primary informal caregivers.

Potential care recipients were initially identified through community health records. Their disease diagnoses and functional status were subsequently verified before their primary informal caregivers were contacted. To obtain diverse caregiving experiences, sampling considered the caregiver–care recipient relationship, urban or rural residence, severity of disability, severity of dementia, and composition of cardiometabolic diseases [35].

#### 2.2.2. Inclusion Criteria

Inclusion criteria for care recipients: (1) age ≥ 60 years old; (2) diagnosed by a secondary-level or higher hospital, with medical documentation, as having at least two cardiometabolic chronic diseases. Based on previous research and relevant International Classification of Diseases, Tenth Revision (ICD-10) diagnostic codes, eligible cardiometabolic diseases were restricted to hypertension, diabetes mellitus, coronary heart disease, stroke, and dyslipidemia [36]; (3) had disability, assessed using 12 items covering activities of daily living (ADL) and instrumental activities of daily living (IADL). Each item was scored from 1 to 4 according to whether the activity could be performed without difficulty, with difficulty but independently, with difficulty and assistance, or not at all. Higher total scores indicated greater functional impairment. Scores of 0–12 represented no impairment, 13–24 mild impairment, and ≥25 moderate or greater impairment. Care recipients with a score of ≥13 were included in this study [37]. (4) had a clinically confirmed diagnosis of dementia made by an appropriate specialist at a secondary-level or higher hospital, as documented in the medical record or diagnostic certificate. (5) lived at home and received ongoing assistance with daily care.

Inclusion criteria for caregivers: (1) Primary informal caregivers (spouses, children, children-in-law, or other relatives) providing care without financial remuneration; (2) Care provision ≥3 days per week with cumulative care duration ≥3 months; (3) Provided informed consent and voluntarily participated in the study.

#### 2.2.3. Exclusion Criteria

Exclusion criteria for care recipients: (1) Admitted to formal care institutions including nursing homes or long-term care facilities; (2) In terminal disease stage or critical condition with unstable vital signs based on the patient’s clinical condition or documented medical diagnosis.

Exclusion criteria for caregivers: (1) Paid caregivers with formal employment relationships; (2) Experienced major life events within the past 3 months that might affect emotional stability during interviews; (3) Diagnosed with malignant tumors, mental illness, or cognitive impairment preventing interview participation.

#### 2.2.4. Sample Size

Sample size was determined by data sufficiency and thematic saturation. Data collection and preliminary analysis ran concurrently; after each interview, recordings were transcribed and preliminarily coded, and emerging meaning units/themes were continuously compared with earlier ones. Saturation was reached when successive interviews yielded no new core themes and no expansion of existing ones. Preliminary saturation was achieved after the 23rd interview; 3 additional caregivers with differing characteristics confirmed saturation, generating no new substantive themes and requiring no modification of the thematic structure. The final sample comprised 26 primary informal caregivers [38].

### 2.3. Data Collection

Interviews were conducted individually, face-to-face, in caregivers’ homes or quiet private community rooms. Researchers explained purpose, procedures, recording, and confidentiality, and obtained written informed consent. Interviews lasted 30–60 min, fully audio-recorded, with one researcher documenting key information and non-verbal expressions. Researchers maintained a neutral stance, avoided leading questions, encouraged free expression, and used clarifying probes; if a caregiver showed strong emotion, the interview was paused and support given, with resumption decided by the participant.

The interview guide was initially developed based on the micro-, mezzo-, and macro-level framework of social ecosystem theory, combined with relevant literature review and research team discussions [34]. Two caregivers meeting inclusion criteria participated in pilot interviews to test question suitability and intelligibility, ensuring comprehensive coverage of relevant topics. The final guide was established after adjusting expression and sequence based on feedback: (1) What feelings have you experienced during the caregiving process? How has caregiving affected you? (2) What have been the most difficult challenges to manage during caregiving? (3) How do you cope with difficulties and stress in caregiving? (4) How do you arrange your leisure time and social interactions? (5) What types of assistance and support do you hope to receive during caregiving? (6) What support or impact do relevant policies have on your caregiving? (7) What are your expectations and concerns regarding your future caregiving life?

### 2.4. Data Analysis

Data collection and analysis were conducted concurrently, guided by the procedural steps of Colaizzi’s seven-step method. Y.Y. and R.W. repeatedly read the transcripts, extracted significant statements related to caregiving experiences, condensed them into meaning units, and developed initial codes. Similar codes were compared and inductively grouped into categories and themes, without using the micro-, mezzo-, or macro-level framework during the initial analysis. The emerging themes were then checked against the original transcripts and refined to ensure consistency with participants’ accounts.

After the thematic structure was established, social–ecological systems theory was applied as a second-stage interpretive framework. The inductively generated themes were mapped onto the micro-, mezzo-, and macro-levels to interpret the relationships among individual caregiving burden, family and community support, and broader institutional and sociocultural factors. Relevant findings that could not be readily accommodated within the framework were retained.

Coding, theme development, and theoretical mapping were conducted by Y.Y. and R.W. X.W. and L.X. reviewed the significant statements, coding logic, thematic structure, and theoretical mapping. When disagreements arose, Y.Y., R.W., X.W., and L.X. returned to the original transcripts and contextual information for further discussion. Unresolved disagreements were referred to M.J. and Y.M. for review, with final decisions based on the support provided by the original data and relevance to the research question. In addition, selected caregivers participated in member checking by comparing the summarized themes with their original interview accounts, and their feedback was used to verify the consistency and credibility of the findings.

### 2.5. Ethical Consideration

This study strictly adhered to medical ethical principles (KJ2024-210-01). Before participation, all subjects received comprehensive information on purpose, procedures, confidentiality, and voluntary participation. All participants provided written informed consent and could withdraw at any time without affecting their medical and care services. All data were anonymized and privacy strictly protected.

### 2.6. Quality Control

Multiple measures ensured rigor. Core team members had systematic qualitative training and interview/analysis experience. Coding and theme refinement were done independently by two researchers then discussed to consensus, reducing subjective bias. Interview data were cross-validated with field observation notes to enhance credibility.

## 3. Results

**Figure 1:**
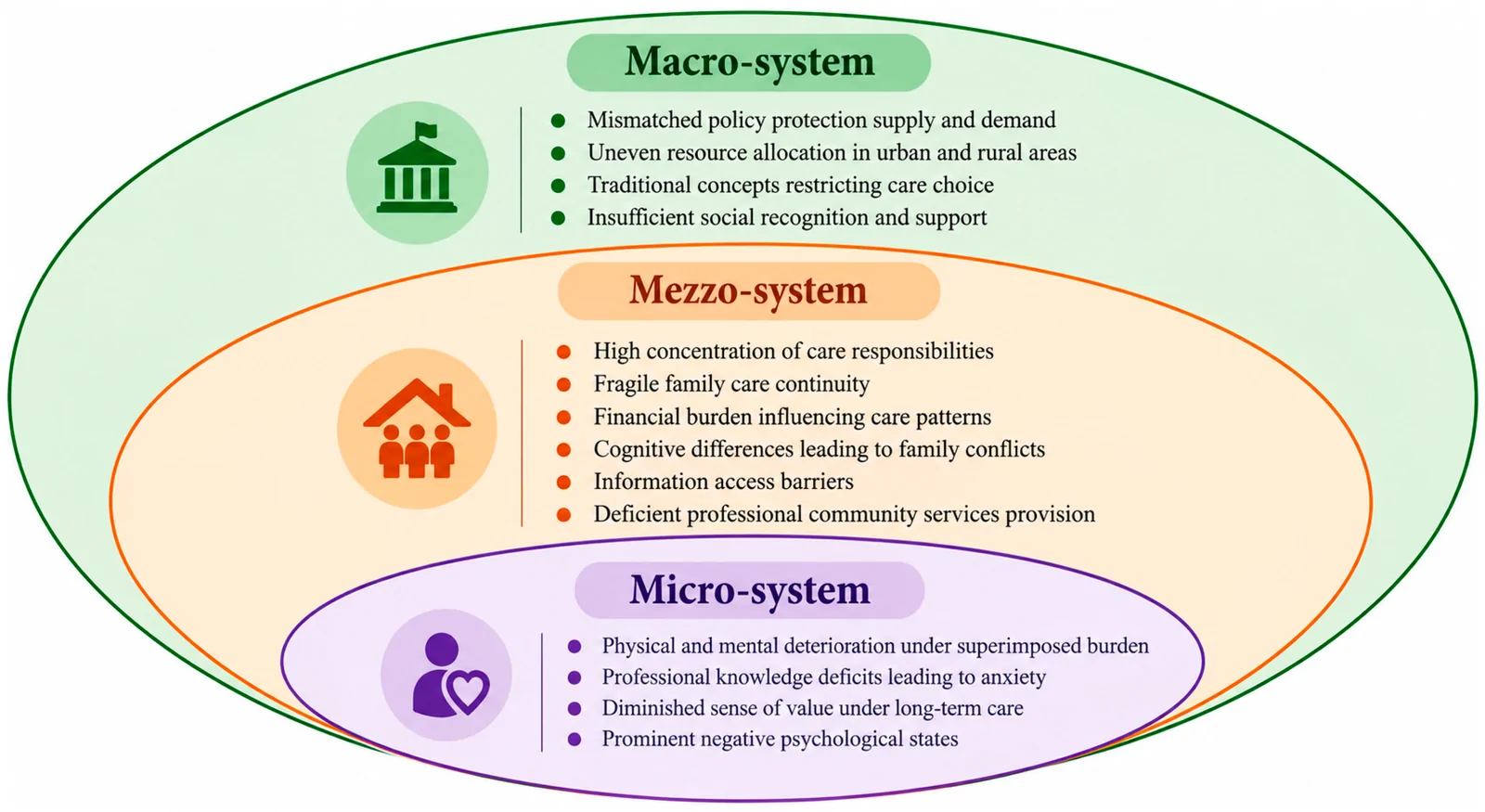
Schematic diagram of the three-layer social ecosystem theoretical framework for informal care burden among older adults living with disability, dementia and cardiometabolic multimorbidity.

Based on the three-tier framework of social ecosystem theory, we coded and summarized all interview transcripts, and systematically sorted out the multi-layer superimposed burden of informal caregivers. Three core themes corresponding to micro, mezzo and macro social systems and a total of fourteen subthemes were extracted from the qualitative data. The overall structural framework of caregiver burden is displayed in Figure 1, while the basic demographic information of the 26 pairs of caregivers and care recipients included in this study is shown in Table 1.

### 3.1. Theme 1: Physical and Psychological Exhaustion and Insufficient Professional Care Competence

#### 3.1.1. Concurrent Deterioration in Physical and Psychological Well-Being Under Cumulative Caregiving Burden

Basic assistance with activities of daily living and the management of concurrent CMM were highly intertwined, resulting in intensive and continuous caregiving demands and a substantial overall physical and psychological burden on caregivers. Spousal caregivers were predominantly older adults who often had chronic health problems or declining physical functioning themselves, which were further aggravated by prolonged caregiving demands. Adult-child caregivers had to balance caregiving with family and work responsibilities, and prolonged exposure to heavy caregiving demands and stress adversely affected both their physical and psychological well-being. The progressive deterioration of caregivers’ own health further increased the difficulty and potential risks of sustaining care.

*C2: “My own health isn’t good either. What would happen if something happened to me?”; C16: “I have to get up every night to check on them. I sleep very poorly, and I often don’t feel well myself.”*

#### 3.1.2. Insufficient Professional Information Intensifying Caregiving Stress

Disability and dementia limited care recipients’ ability to independently manage chronic conditions, requiring caregivers to make repeated judgments about infection prevention, diet, medication assistance, and abnormal health conditions. Although general health information was available, caregivers lacked continuous guidance tailored to specific home-care situations. The absence of clear, actionable guidance, including when to seek professional help, increased care-related uncertainty, difficulty, and anxiety.

*C3: “I don’t even know where to find professional caregiving information.”; C24:“ I don’t know how to prevent urinary tract infections. I dare not administer medications arbitrarily. I can only cook plain congee and noodles, and he keeps getting thinner. I don’t know what else to do.”*

#### 3.1.3. Long-Term Caregiving Constraining Personal Life and Social Participation

The co-occurrence of disability, dementia, and CMM meant that caregiving was not a temporary or episodic responsibility but a prolonged and continuous process with no clearly foreseeable endpoint or period of relief. Both adult-child and spousal caregivers experienced substantial changes in their daily lives. These changes extended beyond simply spending more time on caregiving: caregivers’ personal roles and activities were gradually displaced by caregiving responsibilities, resulting in progressively restricted opportunities for social participation.

*C7: “I also want to have some time of my own.”; C18:“ I used to go to activities, play cards and dance with my friends, but now I can’t go away. I must take care of him at home every day, and there are few people contact me anymore.”*

#### 3.1.4. Prominent Negative Psychological Responses

Fluctuations in care recipients’ functional, cognitive, and health status made caregiving difficult to predict and contributed to persistent negative emotions. Older caregivers expressed concerns and helplessness about sustaining care because of their own health limitations, while adult-child caregivers commonly experienced anxiety, fatigue, and frustration under prolonged caregiving and financial pressure.

*C1: “I take care of [the care recipient] carefully every day, but I don’t know when this will ever end. Sometimes the condition is better, and sometimes it’s worse.”; C11: “Sometimes I just cry.”; C17: “I’m under a lot of pressure every day.”*

### 3.2. Theme 2: Mezzo System–Concentration of Family Caregiving Responsibilities and Mismatch in Community Support

#### 3.2.1. High Concentration of Caregiving Responsibilities

Although some families had other relatives who could potentially provide support, daily care, accompaniment to medical appointments, and management of health-related problems remained concentrated primarily on a single caregiver because of factors such as geographic distance, work commitments, and established patterns of family responsibility. Spouses often assumed caregiving responsibilities because of emotional attachment and a sense of responsibility developed over many years of living together. Influenced by traditional family values, they were also more inclined to provide care themselves rather than burden their children. Adult children became primary caregivers when other family members were unable to assume caregiving responsibilities; geographic proximity, available time, and established family divisions of responsibility further contributed to the concentration of caregiving tasks on one individual.

*C18: “I’m the only one taking care of them.”; C22: “I do everything.”; C25: “They occasionally come by and bring something, but I’m the one who does all the caregiving.”*

#### 3.2.2. Fragility of the Family Caregiving Chain

Population mobility had led some younger adult children to seek employment and development opportunities elsewhere, while other family members were often unable to undertake sustained caregiving for various reasons. Primary caregivers were predominantly older spouses and middle-aged or retired adult children. Many were themselves older and living with chronic conditions. In the absence of substitute caregivers within the family, they had little choice but to continue providing care. Any deterioration in the primary caregiver’s health could therefore jeopardize the continuity of existing caregiving arrangements, leaving the family caregiving chain vulnerable to disruption.

*C20:“ The other children are not in Xinjiang, so they provide little care and minimal financial support.”; C24: “They all work elsewhere and live far away.”; C26:“ If I don’t care for him and there is no one else to take care of him.”*

#### 3.2.3. Financial Burden Shaping Care Arrangements

Ongoing care-related expenditures placed considerable financial pressure on families and became an important factor shaping their choice of caregiving arrangements. Care recipients often had limited financial resources that were insufficient to cover substantial medical and care-related expenses, while the cost of market-based professional care services exceeded what many families could afford. Financial constraints therefore represented a major reason why families did not purchase external care services and instead relied on informal care provided within the family. For some adult-child caregivers, caregiving responsibilities also affected employment and income, further increasing financial pressure. These economic constraints left families with little choice but to rely on relatively low-cost forms of care.

*C5:“ Hiring a nanny for a few hours daily care costs 4000–5000 yuan per month. We don’t have that much money.”; C15: “The pension is only a little over one thousand yuan. It’s not enough.”*

#### 3.2.4. Differences in Perceptions Contributing to Family Conflict

Family members who were not directly involved in caregiving often had difficulty understanding the intensity and risks associated with caring for an older adult with disability and dementia and concurrent CMM. They sometimes underestimated the primary caregiver’s contributions or questioned caregiving decisions and the allocation of expenses. Primary caregivers, already physically and psychologically exhausted by prolonged caregiving, often felt that their efforts were not understood or acknowledged. These differences could lead to emotional confrontation and arguments, contributing to strained family relationships.

*C4: “We don’t even keep in contact anymore.”; C14: “Every time we talk, they criticize this and that. They don’t really help with the caregiving or contribute much money. Whenever we get in touch, we end up arguing.”; C19: “None of them understands how much I’ve put into this.”*

#### 3.2.5. Insufficient Provision and Accessibility of Professional Information

Most caregivers lacked professional knowledge relevant to caring for older adults with CMM. The information sources available to them in everyday life were fragmented, and the information provided was often poorly matched to the complexity of their actual caregiving situations. During in-person healthcare encounters, complicated consultation processes and limited opportunities to obtain detailed guidance made it difficult for caregivers to receive continuous and precise professional advice on a routine basis. When available information could not be readily translated into concrete and actionable strategies for home care, caregivers had to integrate and interpret the information themselves, further increasing the professional demands placed on them.

*C3: “I don’t even know where to find professional caregiving information.”; C13: “There are so many things I just don’t know.”; C20: “I need professional knowledge about how to care for these conditions.”*

#### 3.2.6. Insufficient Provision of Professional Community-Based Services

Community-based services mainly focused on basic daily care, with limited professional support tailored to older adults with disability and dementia and concurrent CMM. Caregivers reported unmet needs for medication management, physiological monitoring, nutritional guidance, and safety risk assessment, indicating a mismatch between available services and actual care needs.

Limited community healthcare resources and infrequent home visits further restricted access to professional support. Community healthcare professionals also had difficulty providing clear guidance on complex issues such as polypharmacy, diet–medication interactions, and early recognition of complications. Consequently, caregivers often relied on their own experience to manage care-related problems, limiting the role of community services in reducing caregiving burden.

*C6: “They don’t have the expertise. How would they know how to deal with these things?”; C8: “They only do simple things like measuring blood pressure or blood glucose.”; C17: “The community healthcare staff are all very busy.”; C18: “Sometimes they come to the home, take a quick look, and measure blood pressure.”; C24: “The community doctor can’t really explain what to do either. They just tell us to take [the care recipient] to the hospital.”*

### 3.3. Theme 3: Macro System–Policy Protection Gaps and Sociocultural Constraints

#### 3.3.1. Mismatch Between Policy Support and Care Needs

Caregivers’ access to long-term care–related policy support was influenced by policy awareness, application and assessment procedures, and the services provided. Limited access to policy information and complicated procedures increased the time and effort required to obtain support, making it difficult to translate available policy resources into practical assistance for daily care.

Meanwhile, gaps remained between available services and actual care needs. For example, long-term care insurance mainly covered basic assistance with activities of daily living, while professional needs such as medication management and nutritional support for older adults with disability and dementia and concurrent CMM were less adequately addressed. Families therefore continued to bear substantial caregiving responsibilities.

*C4: “I don’t understand these things.”; C17: “The application process was really complicated. We had to prepare so many documents and take [the care recipient] to the hospital for an assessment by the department responsible for it.”; C23: “We don’t know about these policies.”*

#### 3.3.2. Uneven Resource Allocation in Urban and Rural Areas

Awareness and reach of relevant policies were more limited in rural areas. Families often had insufficient information about available support programs and application procedures, making it difficult for policy support to effectively reach those in need. Healthcare resources were also relatively limited in rural areas, where accessing healthcare and home-based services was more difficult than in urban areas. For families living far from healthcare facilities and caring for older adults with restricted mobility, accessing services required additional transportation, accompaniment, and time. These barriers made it difficult to meet the ongoing and professional care needs associated with CMM and further increased caregivers’ difficulties in obtaining external support.

*C11: “We hardly go out now. The hospital is far away, and because he can’t move around easily, taking him to see a doctor is extremely difficult.”; C13: “With many policies, we only hear about them from other people long after they’ve been introduced.”; C16: “It’s not easy even for community staff to provide home visits.”*

#### 3.3.3. Traditional Concepts Restricting Care Choice

Family ethics, emotional bonds, and a sense of familial responsibility permeated the long-term caregiving process. Some caregivers preferred to provide care personally and regarded caring for a spouse or parent as a responsibility that family members should fulfill. Such emotional attachment and perceived responsibility therefore had a dual role in the caregiving process. At the same time, some caregivers expressed concerns and negative perceptions regarding institutional care or market-based care services. Even when experiencing substantial caregiving difficulties, they were reluctant to choose institutional care or seek external assistance as a substitute for family caregiving. In this way, traditional family values contributed to maintaining and reinforcing a family-centered model of care.

*C4: “How could a nursing home take care of [the care recipient] as well as family can? I don’t think they would care for people wholeheartedly.”; C17: “I think it’s better if I provide the care myself.”; C21: “I’m afraid other people might dislike him and deliberately mistreat him.”*

#### 3.3.4. Insufficient Social Recognition and Support

Public understanding of caregiving for older adults remained somewhat limited, with caregiving often perceived simply as assistance with basic activities of daily living. Such perceptions may overlook the professional knowledge, intensity, and risks involved in caring for older adults with disability and dementia and concurrent CMM, leaving caregivers’ time commitment, contributions, and caregiving work insufficiently recognized by society. Meanwhile, social support services specifically tailored to this population remained underdeveloped. Targeted caregiving skills training, routine psychological support, and periodic respite care were relatively limited, leaving caregivers with insufficient opportunities to obtain practical and emotional support outside the family.

*C7: “I wish there were somewhere I could go just to talk, somewhere with people who could understand how I feel.”; C18: “Other people think you’re just looking after someone and that it’s easy. Only those who’ve actually done it know what it’s like. I’m so exhausted that I just want a chance to catch my breath.”*

## 4. Discussion

### 4.1. Micro-Level: Reducing Caregiving Stress and Supporting Caregiver Well-Being

From a micro-level perspective, the key issue is not simply whether caregivers possess sufficient disease-related knowledge, but how they coordinate multiple caregiving demands, changes in the care recipient’s condition, and their own limited time and energy when disability, dementia, and CMM coexist [39]. Previous research on dementia with multimorbidity has largely examined dementia care and chronic disease management separately, with limited attention to how cognitive impairment and multimorbidity jointly shape family caregiving [16]. This issue is particularly relevant to the present population: functional dependence increases the need for assistance with daily activities, dementia limits patients’ ability to understand and follow health-management requirements, and CMM requires long-term monitoring and management. When these conditions coexist, caregiving difficulty is more likely to arise from continuous coordination and decision-making than from deficits in any single caregiving skill [18]. Thus, the burden may be better understood as a mismatch between patients’ complex care needs and the family’s available time, energy, and information resources.

Accordingly, nursing support should move beyond conventional knowledge-based education. A 2025 randomized trial among dementia caregivers in Hong Kong showed that self-care, behavioral management, behavioral activation, mindfulness, and support-group components affected different caregiving outcomes, suggesting that caregiver support should be tailored to specific needs rather than relying on education alone [40]. Similarly, a 2025 Beijing study integrating iSupport into family physician services improved caregivers’ learning behaviors but found no significant between-group improvements in burden, quality of life, or social support; implementation challenges included limited time and poor fit between standardized content and individual needs [41]. For families caring for older adults with disability, dementia, and concurrent CMM, nurses could therefore develop a prioritized caregiving problem list during initial assessment and after major changes in condition. Guidance could focus on one or two immediate problems at a time, such as skin and continence care, feeding difficulties, dementia-related behaviors, chronic disease monitoring, or management of abnormal symptoms, with follow-up to confirm whether the recommendations are feasible in practice. Professional boundaries should also be made explicit so that families are not given additional responsibility for clinical judgments beyond their capacity.

Caregivers’ own health also requires attention. A 2025 systematic review of caregiver burden trajectories showed that burden changes over time and that solo caregiving, greater functional limitations of the care recipient, and lower caregiver self-efficacy are associated with persistently high burden [42]. Therefore, restricted rest, reduced personal life, and concerns about the sustainability of caregiving should be treated as early signs that family care may be approaching an unsustainable level. During follow-up, community nurses or family physicians could assess caregivers’ sleep, chronic disease control, emotional status, opportunities for short breaks, and whether another person could take over if the caregiver became ill. Older spouses, sole caregivers, and those with worsening health or no substitute caregiver should receive higher-priority follow-up and earlier assessment for family sharing and external support [43].

Digital technology may provide additional assistance, but “smart” solutions should not be treated as an end in themselves. The Beijing iSupport study also showed that digital interventions may be constrained by older caregivers’ digital adaptability, limited time, and insufficient personalization [41]. For this population, low-burden digital tools may be more appropriate—for example, tools preconfigured by professionals to provide individualized care reminders, record questions for consultation, and enable rapid contact with community professionals, without requiring caregivers to enter large amounts of health data [44]. Future intelligent caregiving assistants could similarly focus on information organization, reminders, risk alerts, and clear referral pathways. Their role should be to reduce unnecessary decision-making burden, protect caregiver well-being, and support the sustainability of long-term care.

### 4.2. Mezzo-Level: Family Sharing and Community-Based Support

This study found that caregiving responsibility was often concentrated in a single family member, influenced by smaller family size, labor migration, and limited household income. Whether the primary caregiver was a spouse or an adult child, the major source of pressure was not simply the duration of care, but the absence of a stable substitute. When caregivers needed medical care, rest, or time for personal matters, there was often no one available to take over; when the care recipient’s condition changed, there was also limited opportunity to share decisions. Solo caregiving therefore concentrated physical work, time demands, and risk-related decision-making in one person. Previous longitudinal studies have similarly identified solo caregiving as an important correlate of persistently high burden [45]. Our findings further suggest that relatives living far away, providing limited financial support, or poorly understanding the intensity of care may exist within the family network without being readily usable sources of support. Family assessment should therefore go beyond asking whether relatives are available and instead identify who can provide respite, participate in medical communication, or contribute transportation and financial support, translating general “family support” into practical caregiving roles.

At the community level, the key problem was not the complete absence of services, but a gap between what was available and what could actually solve caregivers’ problems. Blood pressure or glucose checks and routine follow-up were useful, but existing services often provided limited guidance when families faced polypharmacy, nutritional problems, recurrent infections, interactions between dementia-related behaviors and chronic disease management, or uncertainty about when referral was needed. A 2025 Beijing cluster randomized trial integrating WHO iSupport into family physician services improved learning behaviors but did not significantly improve caregiver burden, quality of life, or social support, and implementation challenges included poor fit between standardized content and individual needs [41]. By contrast, the long-term U.S. Care Ecosystem trial found that continuous collaborative care and care navigation were associated with reduced caregiver depression and improved self-efficacy. Research on medication management in Finland has likewise emphasized the importance of timely access to professionals familiar with the patient and to updated, individualized information [46]. Taken together, these findings suggest that community support should shift from general education or isolated services toward continuous, problem-oriented support. Stable contact points could provide timely responses to manageable problems, identify a primary professional for complex issues, and connect families with family physicians, specialists, or pharmacists when needed, with follow-up to ensure continuity [47].

External support should also be matched to specific family needs rather than treated as a single category of “social support.” A 2025 scoping review of social-support interventions for dementia caregivers identified peer support, counseling groups, health education, mindfulness, individual therapy, and multicomponent interventions [48], while a 2024 meta-analysis of caregiver–person-with-dementia dyadic interventions found inconsistent overall effects, suggesting that effectiveness depends on matching interventions to patient abilities, caregiver needs, and specific goals [49]. Families lacking substitute caregivers may need respite or replacement care; those lacking decision support may need a stable professional contact; and caregivers with persistent emotional exhaustion may benefit from psychological or peer support [50]. Digital tools can assist with appointment reminders, information confirmation, and remote follow-up, but should be viewed as a means of strengthening continuity rather than as a burden-reduction intervention in themselves [44]. Whether such family sharing, professional contacts, and substitute support can be sustained ultimately depends on institutional accessibility, resource distribution, and families’ willingness to accept outside assistance, linking the meso-level system to the macro-level context.

### 4.3. Macro-Level: Institutional Accessibility and Cultural Influences

At the macro level, caregivers were concerned not only with whether policies existed, but also with whether they were aware of them, could understand and complete application procedures, could tolerate waiting times, and ultimately received services that addressed their most difficult or time-consuming tasks. Pei et al. found in a quasi-experimental study that long-term care insurance (LTCI) implementation was associated with reduced informal caregiving time and improved labor-force participation, although benefits varied across caregiver groups [51]. Li et al., using data from Chinese LTCI pilot programs, also reported an association between formal support and lower burden among adult-child caregivers [52]. These findings suggest that formal long-term care systems may help buffer family burden [53]. However, our findings indicate that policy resources can become meaningful family support only when they are known, accessible, attainable, and aligned with actual caregiving needs. Nurses may therefore serve as a bridge between families and formal systems by assessing whether caregivers are aware of and actually using available long-term care, community, or social services, and by helping them connect with existing resources. Broader reforms, such as expanding reimbursement or restructuring service packages, should be evaluated through larger-scale policy research.

Geographic distribution of resources also affects whether support can be translated into practice. For rural families or those facing transportation barriers, the problem may not be complete service absence but the greater time, travel, and accompaniment required to obtain professional care. These additional costs further restrict the limited time of already overburdened caregivers. Macro-level resource allocation therefore affects micro-level caregiving experiences through meso-level service accessibility. For families caring for people with mobility limitations, living far from health facilities, or struggling with frequent travel, telephone follow-up, appropriate remote communication, and cross-institutional information coordination may reduce unnecessary trips, although remote approaches should not replace clinical problems requiring face-to-face assessment [54]. Policy application and eligibility assessment could also be simplified through online processing, home-based assessment, and faster review where appropriate.

Sociocultural factors further shape whether families are willing to incorporate external resources into care. Chen et al.’s systematic review of 62 qualitative studies found that meaning-making in dementia caregiving involves loss, growth, role value, and relationship adjustment [55]. A 2025 phenomenological study in Chengdu similarly described filial piety as a “double-edged sword”: it may strengthen commitment and resilience, but can also increase guilt and inhibit help-seeking [56]. Nursing support should therefore acknowledge caregivers’ sense of family responsibility and relational meaning. Because the psychosocial states of care recipients and caregivers are interdependent, protecting caregiver well-being is itself important for sustaining the caregiving relationship [35]. Overall, the macro-level system does not directly determine the degree of burden within an individual family; rather, it shapes opportunities for formal services, professional resources, and substitute care, while accessibility and cultural acceptance influence whether those resources actually reach the household [57]. This helps explain why similar caregiving tasks may generate different levels of burden and why the existence of formal resources does not necessarily translate into their effective use.

### 4.4. Integrated Interpretation Based on Social–Ecological Systems Theory

Overall, caregiver burden identified in this study did not arise independently at any single level, but accumulated or was buffered through the ongoing interplay of micro-level care needs, meso-level family and community support, and macro-level institutional and sociocultural environments. At the micro level, complex caregiving tasks constituted the most direct source of pressure; at the mezzo level, the availability of shared family responsibilities and professional support determined whether such pressure could be effectively addressed; and at the macro level, institutional accessibility, resource distribution, and cultural norms further influenced whether external support could actually reach families. Thus, the three levels do not operate in parallel but jointly shape caregivers’ lived experiences [34].

Social–ecological systems theory also helps explain the distinction between the availability of resources and their effective use. Policies, community services, and social support can be translated into meaningful caregiving support only when caregivers are aware of them, can access them, and find them responsive to actual family needs. Information gaps, complicated application procedures, mismatched services, or limited acceptance of external assistance may prevent available resources from effectively reducing caregiver burden [58]. Conversely, when caregiving responsibilities are shared within families, community services can provide continuous professional support, and formal resources are accessible and accepted, support across different levels may work synergistically to reduce the pressure placed on a single caregiver.

Accordingly, support for informal caregivers of older adults with disability and dementia and concurrent CMM should not be limited to improving individual caregivers’ knowledge and skills. Greater attention should be given to coordination across levels, including individualized caregiving guidance and physical and psychological support, shared family responsibilities and continuous community-based professional support, and improved accessibility of institutional and social resources. From a social–ecological perspective, these findings provide a theoretical basis for developing a coordinated and continuous multilevel support system. However, the relationships described above represent an interpretive integration of qualitative data and should be understood as possible pathways rather than confirmed causal relationships.

### 4.5. Limitations

This study has several limitations. First, purposive sampling was used and participants were recruited from a single region, which may limit the transferability of the findings. Second, the data were primarily based on caregiver self-reports and may be subject to recall and social desirability bias; perspectives of care recipients, other family members, and primary healthcare professionals were not included. Third, as a cross-sectional qualitative study without comparison groups, differences in caregiver burden across disease patterns and causal relationships could not be determined. The lack of a standardized classification of dementia severity also prevented comparison of caregiving experiences across different severity levels. Finally, although themes were generated inductively before being interpreted through social–ecological systems theory, the theoretical framework may still have influenced data interpretation. Future multicenter, longitudinal, multi-perspective studies incorporating quantitative methods are needed to further examine these findings.

## 5. Conclusions

Guided by social–ecological systems theory, this study examined the caregiving burden and practical support needs of informal caregivers of older adults with disability, dementia, and concurrent cardiometabolic multimorbidity (CMM) across the micro, mezzo, and macro systems. Caregiver burden did not arise solely from caregiving tasks or individual caregiver characteristics; it developed and accumulated through the continuous interaction of factors across these levels.

At the micro level, basic daily living assistance, dementia-related care, and chronic disease management were closely intertwined, increasing caregivers’ ongoing decision-making demands and depleting their physical and psychological resources. At the mezzo level, concentration of caregiving responsibility within individual family members, together with the mismatch between community support and complex care needs, left individual-level difficulties inadequately shared within families and insufficiently addressed by professional support. At the macro level, institutional accessibility, resource distribution, and sociocultural norms shaped whether external support could be effectively accessed and incorporated into home-based care; family affection and filial expectations could sustain the meaning of caregiving and commitment, yet under some circumstances might also constrain help-seeking outside the family.

Social–ecological systems theory further highlights that the significance of caregiver burden lies not only in the difficulties at each level, but in how factors across levels interact to amplify or buffer that burden. Nursing support should therefore not focus solely on strengthening individual caregivers’ knowledge and skills. Rather, on the basis of clearly delineating the respective responsibilities of family caregiving and professional healthcare, support strategies should simultaneously promote sharing of caregiving responsibilities within families, strengthen continuous professional support, and facilitate the effective translation of available resources into practical family support. Such multilevel support may ultimately enhance the safety and sustainability of home-based care.

## Figures and Tables

**Table 1:** Demographic characteristics of 26 dyads of informal caregivers and corresponding older adults with disability, dementia and cardiometabolic multimorbidity.

Caregiver					Care Recipient						
Code	Sex	Age	Residence	Relationship	Sex	Age	Income	Marital Status	Disability	Dementia	Chronic Diseases
C1	female	81	urban	spouse	male	84	3–5	married	mild	yes	Hypertension, dyslipidemia, CHD
C2	female	70	urban	spouse	male	75	>5	married	moderate	yes	Hypertension, stroke
C3	female	68	urban	spouse	male	68	3–5	married	moderate	yes	Hypertension, diabetes, dyslipidemia
C4	female	81	urban	spouse	male	83	<3	married	moderate	yes	Hypertension, diabetes, dyslipidemia, stroke
C5	female	70	urban	spouse	male	75	>5	married	moderate	yes	Hypertension, diabetes, CHD
C6	male	81	urban	spouse	female	78	>5	married	moderate	yes	Hypertension, diabetes
C7	male	70	urban	spouse	female	70	3–5	married	moderate	yes	Hypertension, diabetes, dyslipidemia, CHD
C8	male	85	urban	spouse	female	83	3–5	married	mild	yes	Hypertension, dyslipidemia, CHD
C9	male	85	urban	spouse	female	82	<3	married	moderate	yes	Hypertension, dyslipidemia, stroke
C10	male	81	urban	spouse	female	78	>5	married	moderate	yes	Hypertension, diabetes
C11	male	71	urban	spouse	female	69	3–5	married	mild	yes	Hypertension, CHD
C12	male	82	urban	spouse	female	80	3–5	married	moderate	yes	Hypertension, dyslipidemia, stroke
C13	male	90	rural	spouse	female	90	<3	married	moderate	yes	Hypertension, diabetes, CHD
C14	male	87	rural	spouse	female	85	3–5	married	moderate	yes	Hypertension, dyslipidemia, CHD, stroke
C15	male	73	rural	spouse	female	71	<3	married	moderate	yes	Hypertension, diabetes, dyslipidemia, CHD, stroke
C16	female	64	urban	child	male	83	3–5	married	moderate	yes	Hypertension, diabetes
C17	female	68	urban	child	male	98	<3	widowed	moderate	yes	Hypertension, diabetes, dyslipidemia
C18	female	57	urban	child	male	90	<3	married	moderate	yes	Hypertension, dyslipidemia, CHD, stroke
C19	female	62	urban	child	female	87	<3	widowed	moderate	yes	Hypertension, diabetes, dyslipidemia, CHD
C20	female	64	urban	child	female	82	3–5	married	moderate	yes	Hypertension, diabetes, CHD
C21	female	47	urban	child	female	78	<3	widowed	moderate	yes	Hypertension, dyslipidemia
C22	female	59	urban	child	female	92	3–5	widowed	mild	yes	Hypertension, CHD
C23	female	55	rural	child	female	90	3–5	widowed	moderate	yes	Hypertension, dyslipidemia
C24	male	58	urban	child	male	84	3–5	married	moderate	yes	Hypertension, diabetes, dyslipidemia, stroke
C25	male	53	rural	child	male	88	<3	married	moderate	yes	Hypertension, dyslipidemia, CHD, stroke
C26	male	50	rural	child	female	82	<3	married	moderate	yes	Hypertension, dyslipidemia, CHD

CHD stands for coronary heart disease; income refers to annual household income, with the unit of ten thousand RMB. “Moderate disability” refers to moderate or above disability (total score ≥25), encompassing both moderate and severe cases.

## Data Availability

The data presented in this study are available on request from the corresponding author due to privacy/ethical restrictions.
